# Integrated metagenomic and metabolomic analyses of the effects of total flavonoids of Rhizoma Drynariae on reducing ovariectomized-induced osteoporosis by regulating gut microbiota and related metabolites

**DOI:** 10.1371/journal.pone.0317832

**Published:** 2025-02-14

**Authors:** Qiuyue Li, Xuelin Wu, Xinyu Niu, Zhangjingze Yu, Shengjie Fang, Xuelei Chu, Jia Zhu, Qinghui Song, Chengzhi Hou, Xu Wei

**Affiliations:** 1 Pharmacological Laboratory of Traditional Chinese Medicine, Wangjing Hospital, China Academy of Chinese Medical Sciences, Beijing, China; 2 Department of Education, Wangjing Hospital, China Academy of Chinese Medical Sciences, Beijing, China; 3 Department of Academic Development, Wangjing Hospital, China Academy of Chinese Medical Sciences, Beijing, China; University of Vermont College of Medicine, UNITED STATES OF AMERICA

## Abstract

TFRD has been widely used in China to treat osteoporosis (OP). However, the specific molecular mechanism of TFRD against OP has not been fully clarified. Our previous studies have also proved that TFRD could attenuate OP and the clinical equivalent dose of 67.5mg/kg/d is the effective dose for TFRD treating OP. Therefore, this study used 67.5mg/kg as the dosage of TFRD in combination with multi omics to investigate the mechanism of action of TFRD in the treatment of OP. The aim of this study was to further elucidate molecular mechanism of TFRD for treating OP based on metagenomic and metabolomic analyses. In this study, hematoxylin-eosin (H&E) staining, micro computed tomography (micro-CT) and bone mineral density (BMD) analysis were used to observe pharmacological effects of TFRD against ovariectomized (OVX)-induced OP. Subsequently, multiomics analysis including metagenomics, untargeted and short chain fatty acids (SCFAs) metabolomics were carried out to identify whether the anti-osteoporosis mechanism of TFRD correlated with gut microbiota and related metabolites. Our results indicate that TFRD could improve the microstructure and density of trabecular bone in OVX rats. 17 differential species, which mainly from *Akkermansia*, *Bacteroides*, and *Phascolarctobacterium* genus, 14 related differential metabolites and acetic acid in SCFAs were significantly altered by OVX and reversed by TFRD. Furthermore, according to results of untargeted metabolomics analysis, it was found that several metabolic pathways such as phenylalanine metabolism, phenylalanine, tyrosine and tryptophan biosynthesis and so on might play an important role in TFRD against OP. In order to further study the relationship between gut microbiota and related metabolites, spearman correlation analysis was used, and showed that gut microbiota such as *Akkermansia muciniphila* might be closely related to several metabolites and metabolic pathways. These findings suggest that TFRD treatment could reduce the effects of OVX-induced OP by altering community composition and abundance of gut microbiota, regulating metabolites and SCFAs. It was speculated that the gut microbiota especially *Akkermansia muciniphila* and related metabolites might play an important role in TFRD against OP, and deserve further study by follow-up experiment. This conclusion provides new theoretical support for mechanism research of TFRD against OP.

## Introduction

Postmenopausal osteoporosis (PMOP) is a type of skeletal disease caused by estrogen deficiency and characterized by osteopenia and bone microarchitecture destruction, which can induce an increased fracture risk [1]. PMOP has problems such as high incidence, hidden disease, many complications, complex mechanism, limited drug treatment and so on. It is of great theoretical value and clinical application prospect to explore new pathogenesis of OP and innovative therapy. Since Swedish scientists first discovered that gut microbiota can increase bone mineral density in mice in 2012, the relationship between gut microbiota and OP has become a hot spot in orthopaedic research in recent years [2]. In 2020, Chevalier et al. researched that warmth can prevent OP by regulating gut microbiota [3]. Wang et al. compared the characteristics of gut microbiota and metabolites in 108 postmenopausal women and found that the abundance and diversity of gut microbiota in OP patients and healthy people were significantly different [4]. Qin et al. conducted a metagenome analysis of gut microbiota in 88 subjects from the First Affiliated Hospital of Zhengzhou University from 2018 to 2019 (28 patients with OP and 60 controls) [5]. Some species decreased (such as *Streptococcus*, etc.), while some species increased (such as Bacteroides, etc.). Therefore, it is of great clinical significance to find drugs treating OP through gut microbiota and to dig into its action mechanism. The study of gut microbiota to improve OP can provide new ideas and strategies for the treatment of OP.

The “gut microbiota-bone” axis is based on the interconnection between the bone and the gut [6]. The gut microbiota can produce a variety of molecules such as enzymes, short-chain fatty acids (SCFAs), and metabolites. Lipopolysaccharide (LPS), the main component of the cell wall of gram-negative bacteria, increases in conditional pathogenic bacteria, continuously produces LPS into the blood circulation, induces an inflammatory response in the body, and reduces a large number of bifidobacterial with the function of protecting the intestinal barrier, making the intestinal the permeability of the tract mucosa increased.

Modern medical research has shown that the imbalance of gut microbiota can lead to abnormal immune regulation, up-regulation of inflammatory factor expression, and promotion of osteoclast differentiation, thereby aggravating bone resorption and inducing the occurrence of OP [7]. In 2021, Liu et al. published research results in the top international journal "Advanced Science", proving that Akkermansia muciniphila promotes bone formation and inhibition in castrated mice [8]. Another study showed that Ackermannia has a bone-protective effect, which is positively correlated with bone mass and can be used as a probiotic to prevent or treat OP [9]. In summary, the gut microbiota can be deeply explored as a potential target for the treatment of OP. However, the mechanism by which the gut microbiota regulates OP is still under study. In the future, the composition of gut microbiota may be able to predict the risk of OP and fractures, and the regulation of the gut microbiota may become a new target for the treatment of OP.

TFRD have definite clinical effect in the treatment of OP, and the curative effect in the treatment of ovariectomized (OVX)-induced OP in rats is remarkable. In recent years, TFRD have been widely used in orthopaedical diseases in Asian countries with pharmacological effects of promoting osteogenesis, anti-inflammatory and antioxidant [10, 11]. Meta-analysis showed that TFRD alone or in combination could significantly improve bone mineral density, and play an important role in the prevention of OP [12]. Furthermore, several studies found that TFRD could protect bone health, promote osteoblast differentiation and maturation, and inhibit osteoclast formation. Shen et al. revealed mechanism of TFRD against OP by osteoclast-related pathway promoting H-type angiogenesis and Song et al. told us TFRD inhibited osteoclastogenesis via up-regulating osteoprotegrin, as well as down-regulating receptor activator of nuclear factor kappa-B ligand (RANKL) expression [13, 14].

It was well known that the bioavailability of flavonoids is very low, flavonoids can be widely metabolized by gut microbiota and gut microbiota plays a key role in the pharmacological activity of flavonoids. Moreover, flavonoids can directly or indirectly regulate the relative abundance and activity of gut microbiota and change the physiological state of intestinal microenvironment [15, 16]. Therefore, it is speculated that the regulation of gut microbiota and related metabolites might play an important role in the TFRD against OP.

This study intends to further clarify its pharmacodynamic mechanism from the perspective of mechanism of action. We selected OVX rats as OP model and investigated the therapeutic effect of TFRD [17, 18]. Subsequently, we used metagenomics, untargeted and SCFAs metabolomics analysis to investigate the specific mechanism of TFRD against OP by hematoxylin-eosin (H&E), micro computed tomography (micro-CT) and bone mineral density (BMD) analysis, and to identify whether anti-osteoporosis mechanism of TFRD correlated with regulation of gut microbiota and related metabolites, so as to provide new theoretical support for mechanism research of TFRD against OP.

## Materials and methods

### Ethics statement

In this study, all methods were performed in accordance with relevant guidelines and regulations, and the reporting in the manuscript follows the recommendations in the ARRIVE guidelines. All animal care and experimental protocols and procedures had been approved by the Laboratory Animal Center Institute of Basic Theory for Chinese Medicine, China Academy of Chinese Medicine Science. Animal certificate number: 110324201102334251.

### Reagents and materials

TFRD (Batch number: 200706) was purchased from Beijing Qihuang Pharmaceutical Co., Ltd (Beijing, China). Alendronate (ALN) enteric-coated tablets (Batch number: 266210501) was purchased from CSPC Ouyi Pharmaceutical Co., Ltd (Shijiazhuang, China). Ethylenediaminetetraacetic acid (EDTA) was purchased from Solarbio (PRC). Hematoxylin-eosin (H&E) was purchased from Baso (PRC).

In our previous work, the dose of TFRD against OP and the chemical composition of TFRD have been analyzed and identified [19] (Zhang et al., 2022), and we confirmed that the clinical equivalent dose of 67.5mg/kg is the effective dose. The equivalent dose for rats was obtained using the formula as follows: 67.5mg/kg/d(Animal equivalent dose) = 10.714mg/kg/d(Human dose) * 6.3(Human Km/Animal Km). The constituents of TFRD was obtained by ultra-high-performance liquid chromatography coupled with electrospray ionization quadrupole time-of-flight mass spectrometry (UHPLC-ESI-Q-TOF-MS) analysis. A total of 191 constituents including naringin, naringenin, kaempferitrin, kaempferol, luteolin, eriodictyol, cianidanol, etc., were then identified [19] (Zhang et al., 2022). Based on preliminary work, this study used 67.5mg/kg as the dosage of TFRD to investigate the mechanism of TFRD treating OP.

### Animal study

60 Sprague-Dawley rats (8 weeks old, 220±10g) were purchased from SPF Biotechnology Co., Ltd (Beijing, PRC). All rats were raised in a standard laboratory condition (temperature: 22±1°C, humidity: 50±10%, 12:12 h light/dark cycle, free specific pathogen), feeding with free drinking water and food. The detailed protocol was approved by the institutional Animal Ethics Committee. After 7-day circumstances adaptation, all rats were randomly divided into sham 1 group, model 1 group, sham 2 group, model 2 group, TFRD group, ALN group (n = 10 per group). Then all rats in the model, TFRD, ALN group underwent bilaterally ovariectomy after anesthesia. For sham group, only the adipose tissue on one side of the ovary was removed and other operations were the same as the surgery groups. All rats were fed normally for 12 weeks after operation. This study was approved by the Ethics Committee of Chinese Academy of Chinese Medical Sciences with the ethics approval number of IBTCMCACMS21-2108-01.

To alleviate suffering, rats were anesthetized intraperitoneally with 4% pentobarbital sodium (40mg/kg body weight) before modeling. At the end of the experiment, rats were again anesthetized intraperitoneally with 4% pentobarbital sodium. Under deep anesthesia, blood was collected from the abdominal aorta, leading to the animal’s demise. After 12 weeks of bilateral ovariectomy, the rats in sham 1 and model 1 group were euthanatized, and then tibia and femur of them were collected for histology, micro-CT and BMD analysis to determine whether OVX rat model was established successfully. Meanwhile, the rats in other four groups were given a 12-week intervention. The rats in TFRD group and ALN group were orally administered with TFRD (67.5 mg/kg/d) and ALN (1 mg/kg/d), respectively. The rats in sham 2 and model 2 group were given the equivalent volume of normal saline. At the end of the experiment, pentobarbital (40 mg/kg) was injected intraperitoneally according to the weight. The tibia, femur, feces and serum of all rats were collected for subsequent histology, micro-CT, BMD, metagenomic, untargeted and SCFAs metabolomic analysis.

### Methods of sacrifice

Rats were deeply anesthetized intraperitoneally with 4% pentobarbital sodium at a dosage of 40mg/kg body weight. Under deep anesthesia, blood was collected from the abdominal aorta, which resulted in the animal’s demise. This method was chosen for its reliability in ensuring a swift and painless end to the animal’s life.

### Histology analysis

The left tibia of rats was fixed with 4% formaldehyde, and then decalcified with 10% EDTA. Subsequently, the samples were embedded in paraffin and sectioned at a thickness of 5-μm. At last, the sections were stained with H&E and photographed with a microscope (Olympus, Japan).

### Micro computed tomography analysis

The right femur of rats was fixed with 4% formaldehyde, and decalcified with 10% EDTA. Subsequently, the femur was scanned by Skyscan 1276 micro-CT (Bruker, USA). The lowest end of the distal femoral growth plate was set as the analysis reference line, and the area with a thickness of 3mm was set as the region of interest (ROI). The 3D image was reconstructed with NRecon software and analyzed with DataViewer, CTvox and CTan software.

#### Bone mineral density analysis

The right tibia of rats was collected for BMD analysis. BMD of rats were detected by Dual energy X-ray absorptiometry and analyzed by Osteocore 3 BMD analysis software.

#### Metagenomics analysis

The microbial genomic DNA was extracted from feces of rats using DNeasy PowerWater Kit (MoBio/QIAGEN, USA) and detected by Quantifluor-ST fluorometer (Promega, USA) and Quant-iT PicoGreen dsDNA Assay Kit (Invitrogen, USA). Then the qualified DNA samples were randomly fragmented to a size with 350 bp by Covaris ultrasonic crusher (Covaris, USA). The DNA library was constructed by end repair, adding A tail, ligating sequencing primer, purification and PCR amplification. The insert size of the library was assessed by Agilent 2100 (Agilent Technologies, Germany), and the effective concentration of the library was accurately quantified by Q-PCR (the effective concentration of the Library > 3nM). Then the DNA library were sequenced on Illumina NovaSeq/HiSeq (Illumina, USA) to obtain raw data according to the manufacturer’s instructions. Subsequently, clean reads was obtained by filtering out low-quality. After single and hybrid assembly, a non-redundant gene catalogue was constructed by clustering. Then non-redundant gene catalogue was predicted using MetaGeneMark, and aligned to NCBI NR library for further species annotation. Based on the gene abundance table, species abundances profile at different taxonomic levels were obtained.

#### Untargeted metabolomics analysis

Serum samples of rats were used for untargeted metabolomics. 100 μL of each sample was transferred into 2 mL centrifuge tubes, added 400 μL of methanol, vortexed and centrifuged to obtain all supernatant and then dried in vacuum. Dissolved samples with 150 μL 2-chlorobenzalanine (4 ppm) 80% methanol solution, and filtered the supernatant through 0.22 μm membrane to obtain the prepared samples. The quality control samples consisted of 20 μ L in each prepared sample for Liquid Chromatography-Mass Spectrometry (LC-MS) detection.

Chromatographic separation was accomplished in a Thermo Ultimate 3000 system (Thermo Scientific, USA) equipped with an ACQUITY UPLC® HSS T3 (150×2.1 mm, 1.8 μm, Waters) column maintained at 40°C. 2 μL of each sample performed for gradient elution was carried out with 0.1% formic acid in water (C) and 0.1% formic acid in acetonitrile (D) or 5 mM ammonium formate in water (A) and acetonitrile (B) at a flow rate of 0.25 mL/min. The linear gradient elution setting was as follows: 0~1 min, 2% B/D; 1~9 min, 2%~50% B/D; 9~12 min, 50%~98% B/D; 12~13.5 min, 98% B/D; 13.5~14 min, 98%~2% B/D; 14~20 min, 2% D-positive model (14~17 min, 2% B-negative model). After chromatographic separation, the Electrospray ionization source experiments were executed on the Thermo Q Exactive Plus mass spectrometer (Thermo Scientific, USA). The setting was as follows: the spray voltage was 3.5 kV and -2.5 kV in positive and negative modes respectively, sheath gas and auxiliary gas were 30 and 10 arbitrary units respectively, capillary temperature was 325°C. The analyzer scanned over a mass range of m/z 81–1 000 for full scan at a mass resolution of 70 000. Data dependent acquisition MS/MS experiments were performed with HCD scan. Dynamic exclusion was implemented to remove some unnecessary information in MS/MS spectra.

#### SCFAs metabolomics analysis

Feces samples of rats were used for metabolomics of SCFAs. Samples were supplemented with 15% phosphoric acid, internal standard (isohexanoic acid) solution and ether, homogenized for 1 min. The supernatant was detected for gas chromatography-mass spectrometer analysis by Thermo TRACE 1310 gas chromatograph system (Thermo Scientific, USA) coupled with a Thermo ISQ LT mass spectrometer (Thermo Scientific, USA). Helium was used as the carrier gas with a flow rate at 1.0 ml/min in an Agilent HP-INNOWAX capillary column (30 m×250 μm×0.25 μm). 1 μL of the analyte was injected in split mode (10:1).

#### Statistical analysis

Data were presented as mean ± standard for at least three separate determinations for each group. Statistical analysis was performed using SPSS version 25.0 (IBM Corp.). The Student’s-t test analysis and one-way ANOVA procedure followed by Tukey test were used to determine whether the difference among groups was statistically significant. Correlations between gut microbiota and differential metabolites were calculated by spearman correlation analysis. *p < 0.05, **p < 0.01, ***p < 0.001, #p < 0.05, ##p < 0.01, ###p < 0.001 were considered to have statistical significance.

## Results

### TFRD treatment could reduce the effects of OVX-induced OP

**Fig 1:**
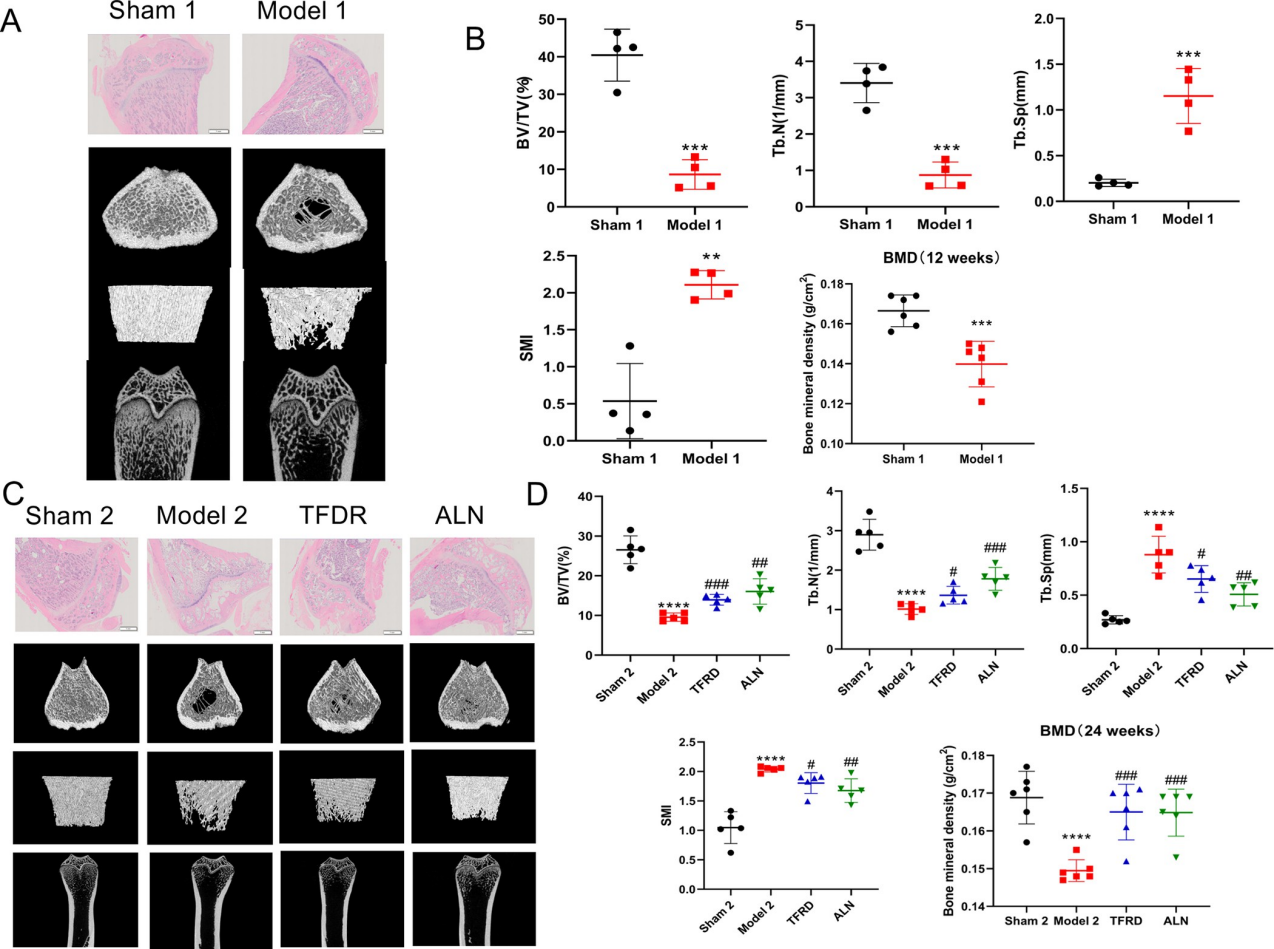
The OVX rat model was successfully established 12 weeks after ovariectomy. (**A**) H&E staining about tibial of rats (n = 3) and micro-CT observation about tibial of rats (n = 5). (**B**) Micro-CT analysis about tibial of rats (n = 5) and BMD analysis about femur of rats (n = 6). After the establishment of OVX rat model (12 weeks), rats were orally administrated TFRD (67.5 mg/kg), ALN (1 mg/kg) or distilled water once a day for 12 weeks. (**C**) H&E staining about tibial of rats (n = 3) and micro-CT observation about tibial of rats (n = 5). (**D**) Micro-CT analysis about tibial of rats (n = 5) and BMD analysis about femur of rats (n = 6). Data were expressed as mean±SD, vs. model group, *p < 0.05, **p < 0.01, ***p < 0.001, vs. the TFRD or ALN group, #*p* < 0.05, ##*p* < 0.01, ###*p* < 0.001.

In this experiment, the rats were ovariectomized to construct OP model. As shown in Fig 1A and 1B, compared with sham 1 group, the number of bone trabeculae decreased and its microstructure was destroyed in model 1 group. In addition, the bone trabecular density of model 1 group was also significantly lower than sham1 group. The result of HE staining, BMD analysis and micro-CT analysis indicated that the OVX rat model was successfully established 12 weeks after ovariectomy. As shown in Fig 1C and 1D, the HE staining, BMD and micro-CT analysis showed TFRD and ALN group improved the bone loss and bone ultrastructure in OVX rats after 12-week oral administration of TFRD and ALN treatment compared with model 2 group. The results indicated TFRD treatment could reduce the effects of OVX-induced OP.

### TFRD treatment altered community composition and abundance of gut microbiota in OVX-induced osteoporosis

**Fig 2:**
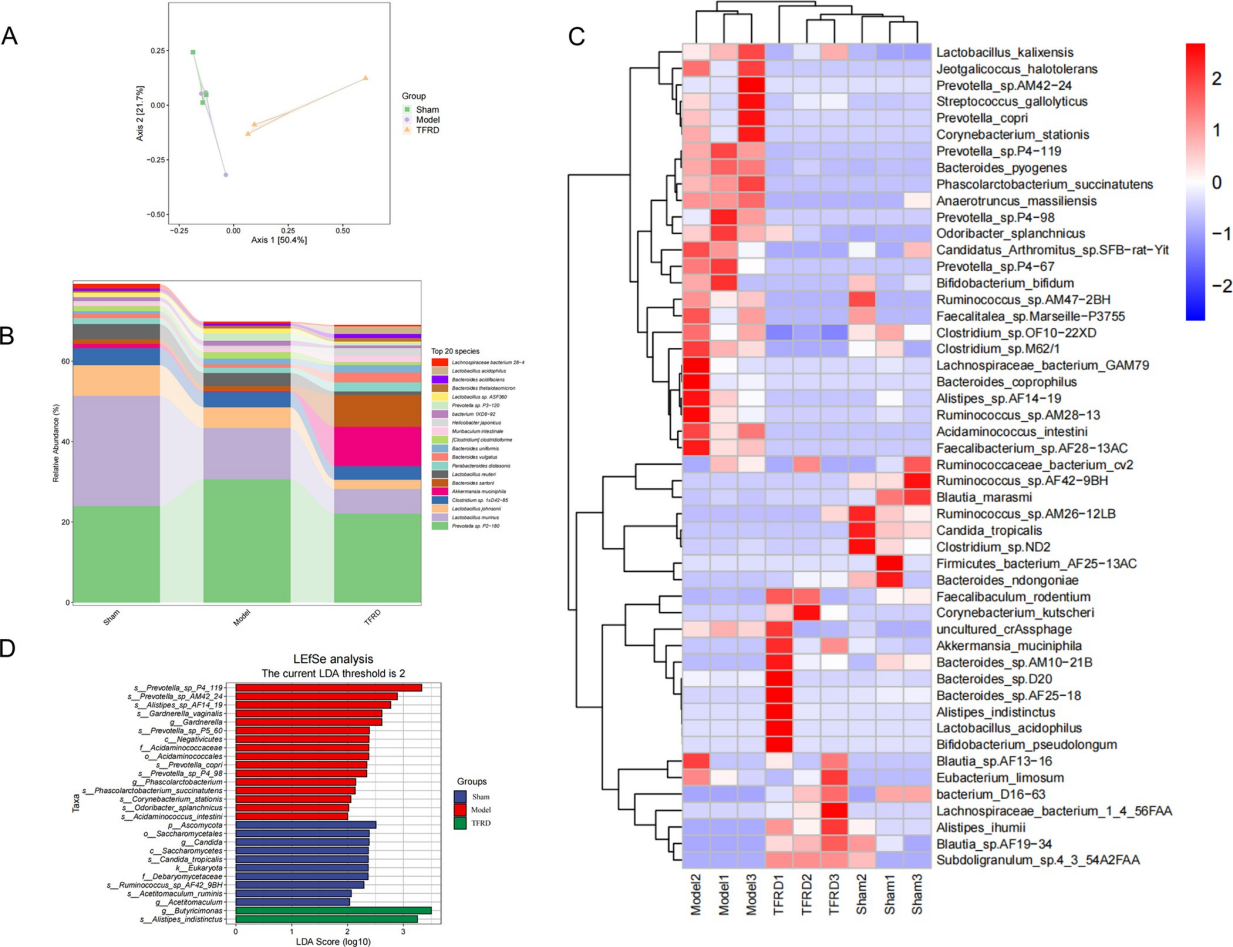
Metagenomics analysis data (n = 3). **(A)** PCoA about sham, model and TFRD groups. (**B**) At the species level, the relative abundances of gut microbiota in sham, model and TFRD groups. (**C**) Heat plot about differential microbiota (top 50). (**D**) LEfSe analysis about gut microbiota in sham, model and TFRD groups.

The principal coordinates analysis (PCoA) showed that there was difference in the microbial community composition of the model group compared with the sham group, while the change in the TFRD group was more significant compared with the model group (Fig 2A). QIIME software was used for Adonis analysis, and 999 permutation tests were conducted to determine whether the differences between groups were statistically significant. The P value obtained from Adonis analysis is 0.013<0.05, indicating statistically significant differences between the groups. In order to analyze the specific changes of gut microbiota, the relative abundance analysis was conducted (top 20). At species level, the relative abundance of *Akkermansia_muciniphila* in TFRD group significantly increased compared with the model group (Fig 2B). Based on criteria of |Log2(Fold_change_value)| > 1 and adjusted P value < 0.05, the differential microbiota was screened at species level, and cluster analysis of which shown in heatmap (Fig 2C, top 50 relative abundance). Combined with the screening results and heat map, it was found that the relative abundance of 17 differential microbiota with OVX interventions was reversed after TFRD treatment, including *Phascolarctobacterium_succinatutens*, *Lachnospiraceae_bacterium_ GAM79*, *Acidaminococcus_intestini*, *Bacteroides_pyogenes*, *Akkermansia_muciniphila*, *Corynebacterium_stationis*, *Faecalibacterium_sp*.*AF28-13AC*, *Jeotgalicoccus_halotolerans*, *Bacteroides_coprophilus*, *Mediterranea_massiliensis*, *and Prevotella_sp*. *P4-98*, *P4-67*, *P4-119*, *AM42-24*, shown in Table 1. The LDA Effect Size (LEfSe) analysis was used to search for the stable differential microbiota between groups. The results of LEfSe showed the relative abundance of *g__Phascolarctobacterium* and *s__Phascolarctobacterium_succinatutens* in model group was significantly higher than sham group and TFRD group (Fig 2D). The results of metagenomics indicated TFRD treatment could alter community composition and abundance of gut microbiota in OVX-induced OP.

**Table 1:** At the species level, the changes of relative abundance of 17 differential microbiota in sham, model and TFRD group.

Name	sham_vs_model_de	sham_vs_model_logfc	TFRD_vs_model_de	TFRD_vs_model_logfc
Prevotella_sp.P4-98	up	6.982	up	7.588
Prevotella_sp.AM42-24	up	6.959	up	7.153
Phascolarctobacterium_succinatutens	up	6.72	up	6.926
Lachnospiraceae_bacterium_GAM79	up	4.293	up	6.647
Prevotella_sp.P4-67	up	6.215	up	6.336
Acidaminococcus_intestini	up	6.277	up	6.245
Corynebacterium_stationis	up	6.07	up	5.917
Prevotella_sp.P5-60	up	5.005	up	5.083
Prevotella_bryantii	up	4.753	up	5.042
Faecalibacterium_sp.AF28-13AC	up	5.107	up	4.995
Ruminococcus_sp.AM28-13	up	3.3	up	4.838
Jeotgalicoccus_halotolerans	up	4.789	up	4.542
Bacteroides_coprophilus	up	4.793	up	4.366
Mediterranea_massiliensis	up	4.126	up	3.841
Prevotella_sp.P4-119	up	2.564	up	2.941
Bacteroides_pyogenes	up	3.113	up	2.768
Akkermansia_muciniphila	down	-1.752	down	-4.741

**Fig 3:**
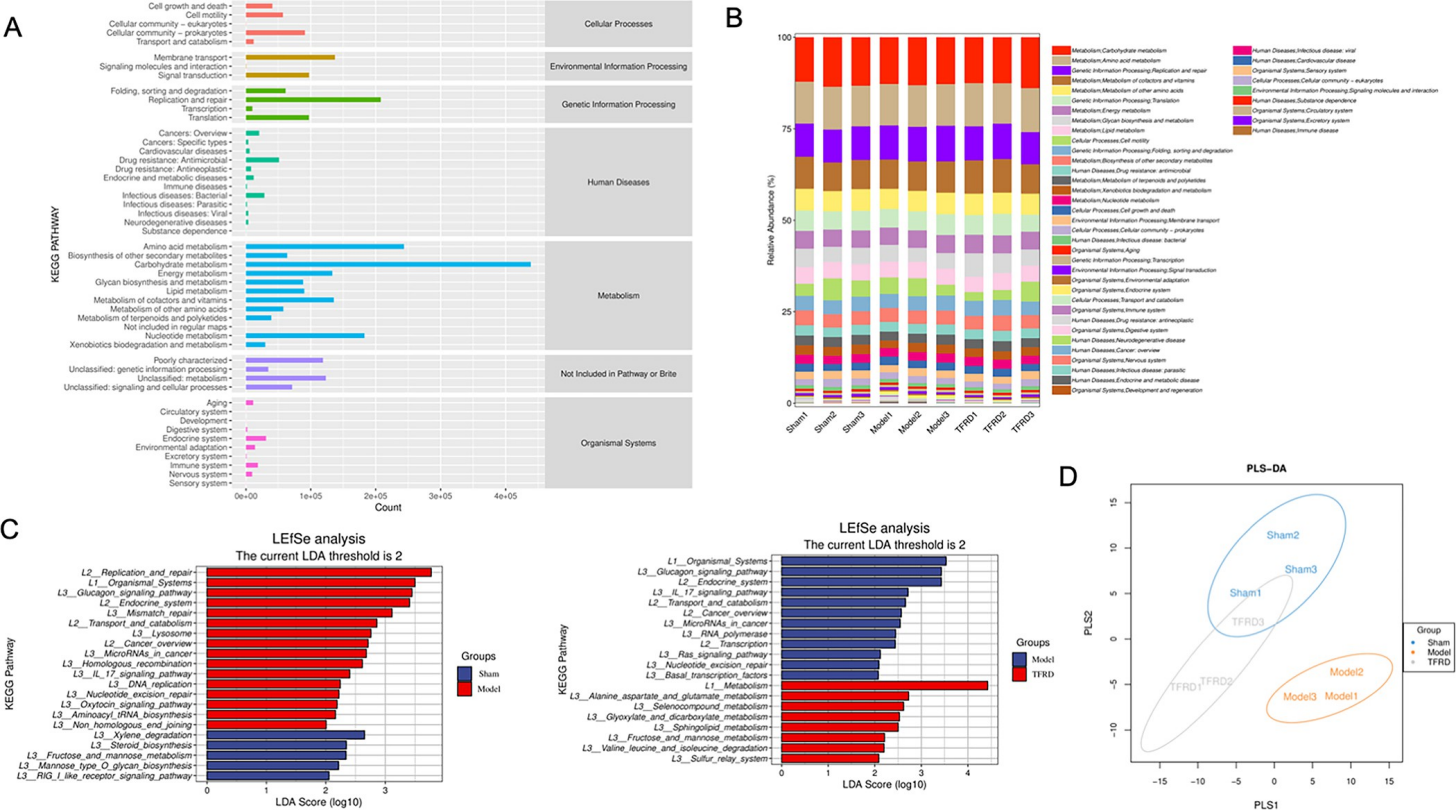
Functional characterization of gut microbiota in TFRD-treated rats with OP. (A) Functional annotation of the Kyoto Ency clopedia of Genes and Genomes (KEGG) orthologs (KOs) is shown as 50 categories of KEGG metabolism pathways. (B) Bar chart of KEGG secondary metabolic pathway composition for each sample: the horizontal axis is arranged according to the sample name, with each bar representing a sample and distinguishing functional group by color. The vertical axis represents the relative abundance of each functional group, and the longer the bar, the higher the relative abundance of that functional group in the corresponding sample. (C) Differentially abundant pathways were identified using LEfSe for KOs in all microbial communities among the TFRD, Model, and Sham groups. (D) Partial least squares-discriminant analysis (PLS-DA) analysis of the KEGG functional pathways shows the intra-group similarity and inter-group difference among the TFRD, Model, and Sham groups.

To further elucidate the functional modules of the fecal microbiome between the three groups, we annotated the multifarious KOs to relevant pathways in KEGG database. As shown in Fig 3A, a total of 50 KOs was screened in these three groups. Fig 3B is the bar chart of KEGG secondary metabolic pathway composition for each sample: the horizontal axis is arranged according to the sample name, with each bar representing a sample and distinguishing functional group by color. The vertical axis represents the relative abundance of each functional group, and the longer the bar, the higher the relative abundance of that functional group in the corresponding sample. To sort out variant functional metabolism pathways, Lefse analysis of KEGG functional profiles was conducted. When comparing the Sham group with the Model group, we found that Replication and repair, Organic Systems, and Glucagon signaling pathway were enriched in the Model group, Xylene deqradation, Steroid synthesis, Fructose and mannose metabolites were enriched in the Sham group. When comparing the Model group with the TFRD group, we found that Organic Systems, Glucagon signaling pathway and Endocrine system were enriched in the Model group, Metabolism, Alanine aspartate and glutamate metabolism, and Selenocompound metabolism are enriched in the TFRD group (Fig 3C). Additionally, there was a clear separation of KOs as revealed by the PLS-DA model among the three groups (Fig 3D).

### TFRD treatment regulate metabolites and related pathways in OVX-induced osteoporosis

**Fig 4:**
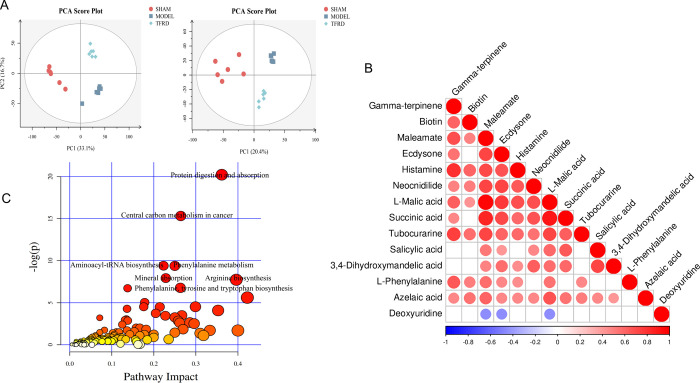
Untargeted metabolomics analysis data (n = 6). (**A**) PCA about sham, model and TFRD groups. (**B**) Heat plot about correlation between 14 differential metabolites. (**C**) Metabolic pathway about differential metabolites in sham, model and TFRD groups.

**Fig 5:**
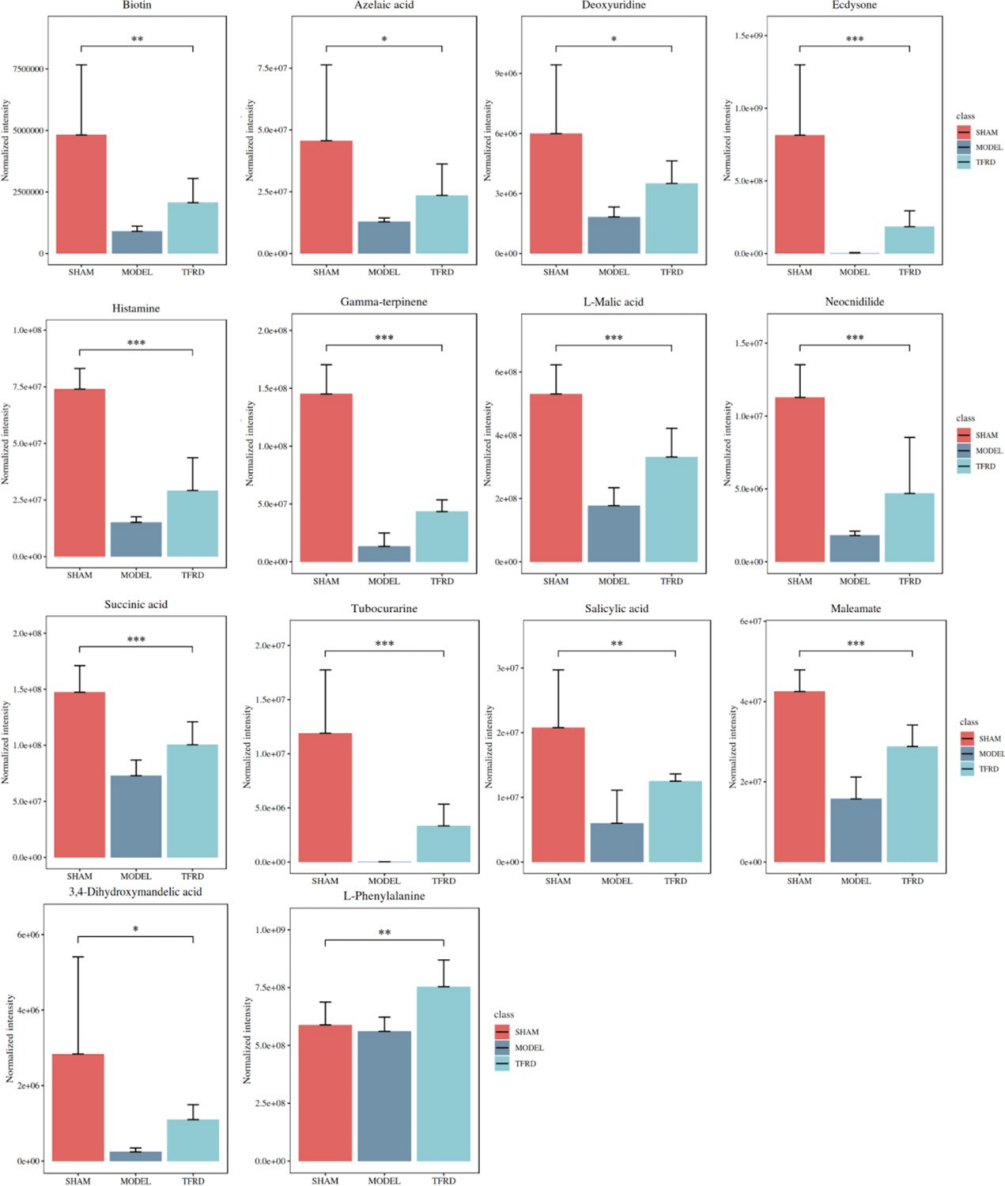
The changes of 14 differential metabolites in sham, model and TFRD group. Data were expressed as mean±SD (n = 6), One-way ANOVA procedure followed by Tukey test was used to evaluate the statistical significance, *p < 0.05, **p < 0.01, ***p < 0.001.

The untargeted metabolomics about serum of rats was carried out by LC-MS to study the metabolic character of TFRD. As shown in Fig 4A, the principal components analysis (PCA) showed the samples among group showed significant difference in the positive and negative mode. Based on criteria of VIP > 1 and p < 0.05, 216 the differential metabolites were screened, and cluster analysis. Correlation analysis was conducted on the 14 differential metabolites in Table 2, and the results are shown in Fig 4B. The correlation analysis results indicate that L-Phenyalalanine have high correlation (P<0.05) with Gamma-terpinene, Biotin, Maleamate, Ecdysone, Histamine, L-Malic acid, Tubocurarine. The 8 differential metabolites pathway was found by metabolic pathway analysis including Protein digestion and absorption, Central carbon metabolism in cancer, Phenylalanine metabolism, Aminoacyl-tRNA biosynthesis, Mineral absorption, Arginine biosynthesis, Phenylalanine, tyrosine and tryptophan biosynthesis and ABC transporters, shown in Fig 4C. As shown in Fig 5 and Table 2, considering the small sample size, other 11 metabolites with reverse trend including Succinic acid, Histamine, Salicylic acid, Biotin, Deoxyuridine, Neocnidilide, Tubocurarine, Azelaic acid, Ecdysone, L-Phenylalanine and 3,4-Dihydroxymandelic acid were also selected as differential metabolites except for Gamma-terpinene, Maleamate and L-Malic acid. The results of untargeted metabolomics indicated TFRD treatment could regulate metabolites and related pathways in OVX-induced OP.

**Table 2:** The changes of 14 differential metabolites in sham, model and TFRD group.

Name	VIP	p.value	Tukeys HSD
Gamma-terpinene	1.392570825	2.50E-09	MODEL-SHAM; TFRD-MODEL
Biotin	1.244967001	0.00437288	MODEL-SHAM;
Maleamate	1.186820731	1.61E-06	MODEL-SHAM; TFRD-MODEL
Ecdysone	1.159940656	0.000477135	MODEL-SHAM;
Histamine	1.345676879	9.99E-08	MODEL-SHAM;
Neocnidilide	1.14603809	4.09E-05	MODEL-SHAM;
L-Malic acid	1.272011698	8.14E-06	MODEL-SHAM; TFRD-MODEL
Succinic acid	1.364330632	3.69E-05	MODEL-SHAM;
Tubocurarine	1.148841128	0.000113794	MODEL-SHAM;
Salicylic acid	1.054348091	0.002405445	MODEL-SHAM
3,4-Dihydroxymandelic acid	1.032052275	0.027278675	MODEL-SHAM
L-Phenylalanine	1.410722026	0.006148879	TFRD-MODEL
Azelaic acid	1.03447778	0.028287352	MODEL-SHAM
Deoxyuridine	1.004110563	0.012568633	MODEL-SHAM

### TFRD treatment increased content of SCFAs in OVX-induced osteoporosis

**Fig 6:**
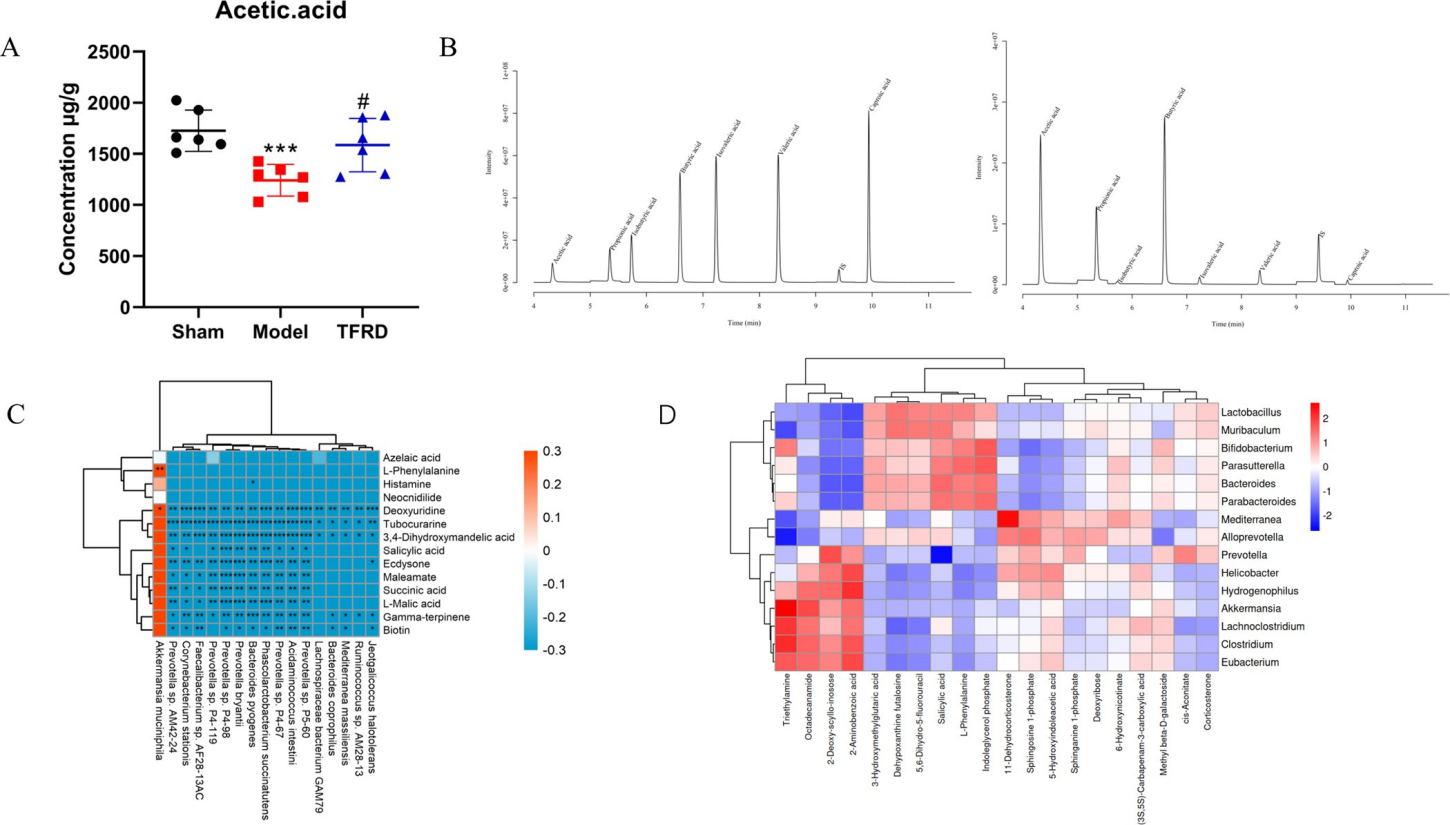
**(A)** The content of SCFAs in sham, model and TFRD group (n = 6). (**B**) Sample and mixed TIC diagrams of SCFAs. Data were expressed as mean±SD, vs. model group, *p < 0.05, **p < 0.01, ***p < 0.001, vs. the TFRD or ALN group, #*p* < 0.05, ##*p* < 0.01, ###*p* < 0.001. (**C**) Correlation of gut microbiota reversed by TFRD intervention wtih differential metabolites was analyzed by Spearman correlation analysis. Heatmap shows the value of correlation coefficient between gut microbiota and differential metabolites. *, **, *** indicate the correlation significance (*p* < 0.05, *p* < 0.01 and *p* < 0.001 respectively). Red represents positive correlation and blue represents negative correlation. **(D)** Microbiome-metabolome interaction probabilities via mmvec predict strong associations between lipidmetabolites with Prevotella, Lactobacillus, Bacteroides, Akkermansia and other top 15 clades.

In this experiment, it was found that TFRD treatment had an effect on the gut microbiota and metabolomic changes of OVX rats. Previous studies found that SCFAs, one of the most important metabolites of gut microbiota, effectively regulated osteoclast metabolism and played an important role in the prevention and treatment of metabolic syndrome. In order to study the mechanism of TFRD against OP, the content of SCFAs was detected in feces. As shown in Fig 6A, compared with sham group, the concentration of acetic acid decreased in the OVX group. There was significant difference between the OVX group and TFRD treatment group in the amount of acetic acid (p <0.05). Therefore, the acetic acid of gut microbiota in rats decreased significantly after ovariectomy, while increased significantly after TFRD treatment, indicating TFRD treatment could increase content of SCFAs in OVX-induced OP.

### Correlation analysis of gut microbiota and related metabolites

In present study, spearman correlation analysis and mmvec analysis were used to further analyze relationship between gut microbiota and related metabolites. As shown in Fig 6C, it was found that 17 differential species reversed by TFRD were negatively or positively associated with at least one differential metabolites. 10 differential species were negatively correlated with Succinic acid. In addition, *Akkermansia muciniphila* were positively correlated with L-Phenylalanine. According to results of the previous metabolic pathway analysis, it was found that L-Phenylalanine and Succinic acid both enriched in Phenylalanine metabolism, a key pathway for TFRD to regulate bone metabolism. The findings using mmvec align well with our initial analysis, confirming the significant presence of species such as Akkermansia muciniphila, Prevotella, and so on. As shown in Fig 6D. Both the two methods highlight L-phenylalanine as a metabolite with a relatively high probability of co-occurrence and its close relationship with these two bacteria. In conclusion, gut microbiota such as *Akkermansia muciniphila* might be closely related to phenylalanine metabolism.

## Discussion

In this study, the efficacy and mechanism of TFRD on OVX-induced OP was explored. HE staining, micro-CT and BMD analysis were used to observe pharmacological effects of TFRD against OVX-induced OP, and the results of which indicated TFRD could improve the bone loss and bone ultrastructure in OVX rats. Subsequently, multiple omics analysis including metagenomic, untargeted and SCFAs metabolomics were used to uncover the mechanism of TFRD against OP. The multiomics analysis revealed that TFRD regulated changes of gut microbiota, metabolites and SCFAs in OVX-induced OP. Furthermore, according to results of untargeted metabolomics analysis, it was found several metabolic pathways such as phenylalanine metabolism, phenylalanine, tyrosine and tryptophan biosynthesis and so on might play an important role in TFRD against OP. At last, spearman correlation analysis showed that gut microbiota such as *Akkermansia muciniphila* might be closely related to phenylalanine metabolism.

The OVX model, a classical PMOP model, was selected in this experiment [20]. As expected, BMD of rats significantly decrease after ovariectomy. In addition, the results of HE staining showed the bone trabeculae of rats were thinner the bone and sparser arrangement in model group compared with sham group, indicating OVX-induced OP model was successfully established. After 12-week TFRD treatment, the reduction of bone mass was significantly improved. Nowadays, microstructural determinants are necessary in assessing the true impact of treatment on trabecular bone quality [21]. Therefore, besides HE staining and BMD analysis, micro-CT analysis has also been used in pharmacodynamic evaluation of TFRD against OP. The results of the micro-CT showed that the morphometric parameters (BV/TV and Tb.N) of the trabecular bone at the distal femur were improved in the OVX rats in response to 12-week TFRD treatment. Furthermore, the TFRD treatment decreased the OVX-induced changes of the Tb.Sp and SMI, indicating that the deterioration of trabecular bone microstructure was significantly improved. Previous studies showed that TFRD could increase number and density of bone trabeculae, and improve the morphology of bone tissue and the biomechanical strength of osteoporotic rats, which is consistent with our above experimental results [22].

The gut microbiota has been verified to be closely associated with OP [23]. In this study, gut microbiota in the feces of rats was analyzed by metagenomics to identify relationship between TFRD against OP and gut microbiota. PCoA results showed that the microbial community in OVX group was different from that in sham group. The gut microbiota composition changed after TFRD treatment. In addition, studies have confirmed that flavonoids can regulate the composition of human gut microbiota [24]. Therefore, PCoA results indicated that ovariectomy caused gut microbiota disorder, and TFRD could affect bone metabolism by altering the composition and distribution of gut microbiota. At species level, after TFRD treatment the abundance of *Phascolarctobacterium_succinatutens*, *Lachnospiraceae_bacterium_GAM79*, *Acidaminococcus_intestini*, *Bacteroides_pyogenes*, *Corynebacterium_stationis*, *Faecalibacterium_sp*.*AF28-13AC*, *Jeotgalicoccus_halotolerans*, *Bacteroides_coprophilus*, *Mediterranea_massiliensis*, *and Prevotella_sp*. *P4-98*, *P4-67*, *P4-119*, *AM42-24* were decreased, while *Akkermansia_muciniphila* was significantly increased. Several studies have found gut microbiota at genus level such as *Phascolarctobacterium*, *Faecalibacterium*, *Bacteroides* and *Prevotella* were increased in the osteoporotic animals and people by 16S rRNA sequencing [25–27]. *Bacteroides_pyogenes* and *Bacteroides_coprophilus* belong to *Bacteroides* which is considered to associate with reduced BMD and T-score at lumbar spines [28]. *Akkermansia muciniphila*, a symbiotic bacterium of the mucus layer, is considered to be a promising probiotic and play a role in preventing bone metabolic disorders because of its properties of anti-inflammatory and reducing bone destruction [9, 29, 30]. A study has found that transplantation of child-derived fecal bacteria with live *A*. *muciniphila* or *A*. *muciniphila*-derived extracellular vesicles (AmEVs) into osteoporotic mice increased osteogenic activity and inhibited osteoclast formation [8]. In addition, *A*. *muciniphila* appears to have therapeutic effects against osteoporotic complications. We found that transplantation of *A*. *muciniphila* promotes H-type angiogenesis and fracture healing by alleviating systemic inflammation in mice [31]. Collectively, these results show that *A*. *muciniphila* can maintain bone mass and strength through multiple pathways, providing a new perspective for the management of osteoporosis. Therefore, TFRD could reduce the effects of OVX-induced OP by altering community composition and abundance of gut microbiota.

It was found that alterations of gut microbiota and metabolites in feces and serum were responsible for the occurrence and development of OP [32]. In this study, untargeted metabolomics analysis in serum was carried out to explore the effect of TFRD on metabolism of OVX-induced OP. PCA results showed that there were significant differences in metabolites between the sham group and model group. The serum metabolites under the action of TFRD showed a trend towards the sham group. In addition, our study found 14 differential metabolites including Gamma-terpinene, L-Malic acid, Succinic acid, Maleamate, Histamine, Salicylic acid, Biotin, Deoxyuridine, Neocnidilide, Tubocurarine, Azelaic acid, Ecdysone, L-Phenylalanine, 3,4-Dihydroxymandelic acid under the action of TFRD. The co-polymers formed by recombinant osteoprotegerin and Maleamate can further improve the therapeutic effect of OP and show stronger bone resorption inhibitory activity [33]. It is reported that the uptake disorder of key molecules such as biotin caused by SLC5A6 gene mutation can seriously affect the development of bone, brain and immunity. This kind of population can be improved after biotin supplementation [34]. Osteopontin, a bone matrix protein involved in the regulation of absorption, can be inhibited by D-tubocurarine, and then inhibit OP induced by nicotine [35]. β-Ecdysone has a protective effect on bone and can promote the osteogenic differentiation of bone marrow mesenchymal stem cells [36]. Furthermore, the results of metabolic pathway analysis revealed several metabolic pathways such as phenylalanine metabolism, phenylalanine, tyrosine and tryptophan biosynthesis and so on might play an important role in TFRD against OP. It is reported that *Drynaria roosii* Nakaike may prevent OP by interfering with phenylalanine metabolism and tryptophan metabolism in rats, which also indirectly confirms our experimental results [37]. L-phenylalanine, one of the essential aromatic amino acids with physiological activity, can increase protein synthesis rates of bone tissue [38]. There are studies indicating that Rhizoma Drynariae may prevent osteoporosis by intervening in phenylalanine metabolism in rats [37]. *Akkermansia* is closely related to the biosynthesis of phenylalanine [39]. Research has shown that changes in serum phenylalanine levels may be one of the reasons for bone loss in children with phenylketonuria [40]. Further study showed that oral administration of low-Phehydrolysate (LPH) prevented bone loss, improved bone mechanical properties, and ameliorated the deterioration of trabecular microarchitecture in an ovariectomy-induced bone loss mice model. Moreover, LPH dual-egulated bone remodel-ing in OVX mice by triggering osteogenesis and inhibiting osteo-resorption and inflammation [41]. In addition, tyrosine, produced by the catalysis of phenylalanine, phosphorylation of which affects the interaction of Cbl-PI3K. The elimination of this effect will disturb bone homeostasis and affect osteoclast function and osteoblast proliferation [42]. Succinate functions as an extracellular ligand through binding to its specific receptor on osteoclastic lineage cells and stimulates osteoclastogenesis in vitro and in vivo [43]. Succinic acid acts inside cells, enhancing and maintaining the expression of endogenous pro-inflammatory genes while inhibiting the expression of anti-inflammatory genes [44]. Further study suggest that succinate may directly regulate Hif‐1*α* signaling and proinflammatory cytokine release, thereby affecting the osteogenic differentiation of MSCs [45].

Spearman correlation analysis showed that most of differential metabolites were positively or negatively correlated with gut microbial species, indicating that TFRD against OP might be closely related to the changes of gut microbiota and related metabolites. Interestingly, L-phenylalanine and succinic acid from phenylalanine metabolism are significantly correlated with *Akkermansia muciniphila*, *Phascolarctobacterium succinatutens* and other gut microbiota, which is also the focus of our study. Several studies have found *Akkermansia muciniphila* could attenuate colonic inflammation by regulating tryptophan metabolism [46]. Phenylalanine and tryptophan are essential aromatic amino acid for human body. Therefore, it was speculated that *Akkermansia muciniphila* and phenylalanine metabolism might play crucial roles in TFRD against OP. We also used the mmvec analysis method. Mmvec is a method used for combined analysis of microbiome and metabolome. It mainly considers the co-occurrence probability of microorganisms and metabolites to predict possible interactions between microorganisms and metabolites. The results of pearman correlation analysis and mmvec analysis are consistent, confirming the significant presence of species such as Akkermansia muciniphila, Prevotella, and so on. While Prevotella is a harmful bacterium and AKK is a beneficial bacterium. Therefore, the conclusion of this study is that TFRD may improve the L-phenylalanine metabolic pathway by regulating AKK to improve osteoporosis.

In recent years, SCFA released by gut microbiota has become a hot spot in the research of OP because it plays an important role in regulation of osteoclast metabolism and bone homeostasis [47]. Therefore, besides untargeted metabolomics in serum, SCFAs metabolomics analysis in feces was also carried out in this experiment. In this study, acetic acid of SCFAs in feces decreased significantly after ovariectomy, and TFRD reversed this change. It has been reported that the content of SCFA including acetic acid and butyric acid in feces of OVX-induced rats decreased, which also confirmed our results [48]. Studies have confirmed that flavonoids can stimulate gut microbiota to produce SCFAs, especially acetic acid and butyric acid [49]. *Akkermansia muciniphila* uses mucin secreted by goblet cells as its main carbon and nitrogen sources, and generates SCFA as its energy source through self secreted metabolic enzymes [50]. Research has shown that AKK bacteria can assist in managing metabolic syndrome related indicators by producing various SCFA, demonstrating great potential in anti-aging [51]. Furthermore, according to a new study, *Akkermansia muciniphila* could promote secretion of SCFA including acetic acid, butyric acid and so on [52]. Therefore, TFRD might reduce the effects of OVX-induced OP by increasing SCFA such as acetic acid.

Many epidemiological, in vitro and in vivo studies have consistently shown that flavonoids exhibit significant anti-inflammatory properties in chronic inflammatory diseases such as autoimmune diseases, cancer, diabetes, cardiovascular diseases and neurodegenerative diseases [53]. Of particular note is that several specific flavonoids have been found to have protective effects against asthma [54]. Further research has shown that flavonoids exert anti-inflammatory effects on the respiratory tract by inhibiting the expression of NF—κ B and inflammatory cytokines [55]. This mechanism has been validated by both in vivo and in vitro studies, further highlighting the broad potential of flavonoids in maintaining health.

There are some limitations to the current study that need to be considered. In this research, only integrated metagenomic and metabolomic analyses were carried out, the detailed mechanisms of gut microbiota need to be further explored. We plan to transplant fecal samples from TFRD rats into OVX-induced OP rats and observe the changes of plasma, composition and function of intestinal flora among transplanted rats, TFRD group, normal group and model group and oral administration of *AKK* bacteria to detect changes in metabolites.

## Conclusion

Based on pharmacodynamics, it was found TFRD treatment could reduce the effects of OVX-induced OP. The metagenomics analysis indicated TFRD treatment could alter community composition and abundance of gut microbiota in OVX-induced OP. Of those, the abundance of *Akkermansia muciniphila* significantly increased. The metabolomics analysis showed several metabolites changed, and acetic acid in SCFA increased after TFRD treatment. Spearman correlation analysis and mmvec align indicated gut microbiota such as *Akkermansia muciniphila* might be closely related to several metabolites (including L-Phenylalanine) and metabolic pathways. In this study, it was speculated that gut microbiota especially *Akkermansia muciniphila* and related metabolites (such as L-Phenylalanine) might play an important role in TFRD against OP. The conclusion of this study is that TFRD may regulate the L-phenylalanine metabolic pathway by regulating AKK to improve osteoporosis.

This conclusion provides a new way to further clarify the molecular mechanism of TFRD against OP, but it still needs to be further verified to obtain direct evidence that TFRD regulates gut microbiota to treat OP.

## Supporting information

S1 Table Raw data results of over 500 differential metabolites. (XLSX)

S2 Table Analysis results table by MMVEC. (XLSX)

S3 Table Fig 6C correlation analysis using spearman correlation analysis on the 17 differentially expressed bacterial species in Table 1 and the 14 differentially expressed metabolites in Table 2. (CSV)

S4 Table Fig 6D mmvec analysis of the top 15 differential species (genus level) and related differential metabolites results. (XLSX)

S1 File The composition and abundance distribution tables of each sample at six classification levels: Phylum, class, order, family, genus, and species using QIIME software. (RAR)

## Abbreviations

TFRD: Total flavonoids of Rhizoma Drynariae
OP: osteoporosis
H&E: hematoxylin-eosin
micro-CT: micro computed tomography
BMD: bone mineral density
OVX: ovariectomized
SCFAs: short chain fatty acids
PMOP: postmenopausal osteoporosis
LPS: lipopolysaccharide
TCM: traditional Chinese medicine
RANKL: receptor activator of nuclear factor kappa-B ligand
ALN: alendronate
UHPLC-ESI-Q-TOF-MS: ultra-high-performance liquid chromatography coupled with electrospray ionization quadrupole time-of-flight mass spectrometry
EDTA: ethylenediaminetetraacetic acid
LC-MS: Liquid Chromatography-Mass Spectrometry
PCoA: principal coordinates analysis
LEfSe: LDA Effect Size
